# The General Practitioner1President’s address, delivered before the Canadian Medical Association, at Toronto, June 1, 1910.

**Published:** 1910-11

**Authors:** Adam H. Wright

**Affiliations:** Professor of Obstetrics, University of Toronto


### The General Practitioner[1]

By ADAM H. WRIGHT, B.A., M.D., M.R.C.E. Eng.
Professor of Obstetrics, University of Toronto.

(*Canadian Practitioner and Review*, July.)

IT is supposed by some that the general practitioner will soon become extinct. Although that seemed possible or probable a few years ago in some cities, such as New York, Chicago, and the like, it appears that the pendulum is swinging the other way, and the family physician is now considered a necessity in most homes. There is perhaps no member of an ordinary community who comes more prominently into view than the doctor. He must run the gauntlet of criticisms very varied in character. Sometimes these criticisms are harsh and unjust, but on the whole we have no cause to complain. One of the finest characters ever described was Dr. McClure. How many such there are we know not: but there are a few—perhaps many. We might name one—Gawn Shaw Cleland of Toronto, who "crossed the bar" last January. The Toronto *Globe*, in an obituary article, said re-

---

1. President's address, delivered before the Canadian Medical Association, at Toronto, June 1, 1910.

specting Cleland: "He was loved and respected by his patients and was looked upon throughout the community as another Dr. McClure."

He it was or such as he that Luke Fildes had in view when he painted that great picture, "The Doctor," nineteen years ago. Sir Mitchell Banks, of Liverpool, England, made the following reference to it in 1892: "Of the hundreds of medical men who have stood before that picture I am sure there was not one whose pulses it did not quicken with pleasurable pride, or who left it without thinking that it already had been, and again would be his privilege to fight against pain and suffering and death like his colleague on the canvas. Note where the scene of the picture is laid: not in some rich man's mansion, but in a workingman's cottage. With admirable skill the painter has pitched on the early hour of the morning for the time. . . . The sick child, worn with the raging fever, lies spent and exhausted. Till then the parents have been fighting on with their nursing: soothing, caressing, encouraging their little one, and hoping against hope seems all that is left to them. And there sits their friend—the gentle doctor—watching with them, and still puzzling his brains to think what more he can devise to stay the lamp of life from flickering out. He is no courtly physician, no London specialist, that man (thank God!). He is only a country doctor. But his somewhat rugged face tells of honesty, and common sense, and self-reliance, and gentleness. What more do you want? The men that look like that man, whatever be their business or trade or profession, whatever be their wealth or their social position, I say, of such men is the kingdom of heaven." The original picture is now in the Tate Gallery, London. We do not pretend that the majority of physicians are saints or heroes; but we do contend that the practice of our profession furnishes grand opportunities for good work in the interests of suffering humanity. We are proud to think that in all parts of Canada there are physicians who make the most of such opportunities.

Some may wonder whether Fildes's doctor will continue to exist. We are told that therapeutics is becoming unpopular because there has been in the past, and is now, too much empiricism in our methods of treatment. The all-important subjects among the final branches are diagnosis, prognosis and pathology. It is supposed by some that the "McClure" and the "gentle doctor" will go out of fashion, and that the modern physician will struggle longer and puzzle more over his diagnosis, and, then in a case such as Fildes's sick child, he will turn to the mother with a bland smile on his wise face, and say to her: "Madam, this is really a most interesting case. It has been very puzzling, but I am pleased to be able to say I have made a diagnosis and prognosis. This child has malignant endocarditis and will die in about five or six hours. I can do nothing more for you now, but I shall call in the morning to make a post-mortem examination."

One of the most vexed questions of the present day is the preparation of general practitioners, *i. e.*, methods of medical education. In recent years there have been many discussions on the subject in the British Medical Association. I am glad that our friend, Dr. Connell, of Kingston, will read a paper on the subject at this meeting. The amount of work in all departments of medicine has increased so enormously during recent years that students are bewildered, confused and disheartened. The students of today bolt more, and cram more, and observe less, and think less, than did those of ten to twenty years ago. There seems to be little continuity between the teaching of the primary and the final subjects. In the early years the students are now swallowing pure and applied science in masses too big for their assimilative organs; or, in other words, are largely memorising facts without understanding them. It is believed by many that this unfortunate condition of things exists in many, if not most of the best medical colleges in North America, as well as in the old world. It would appear that the level-headed Britishers are realising the situation more fully than the teachers of any other countries.

Francis Shepherd, of Montreal, in his presidential address before this Association in 1902, referred to certain defects in modern laboratory teaching. There is probably no man on this continent who understands this subject more intimately than he from two standpoints—the scientific and the practical. He expressed the opinion that in many of our modern hospitals with their laboratories "students are not taught to observe so carefully the evident symptoms of disease, and are becoming mere mechanics. . . . The higher and more intellectual means of drawing conclusions by inductive reasoning are almost neglected."

On the other hand we have scientists who think that such ideas are entirely wrong and not even worthy of consideration. Some of our advanced educationalists are even growing a little tired of Johns Hopkins, because those Baltimore men still stick to the old-fashioned idea that the student should be encouraged to observe and think and reason. We are told that they hope soon to be able to manufacture machine-made physicians and surgeons who will be vastly superior to the home-made article.

As a matter of fact, the differences between the schools of thought commenced many years before Shepherd sounded his note of warning. About fifteen years ago the late Sir George Humphry, Professor of Anatomy, Cambridge University, in an address delivered in Oxford, spoke as follows about methods of teaching medicine: "There is too great a mass of facts heaped on the memory and too little reflection on them. . . . The sciences of physiology and histology have become, and those of pathology and anatomy are becoming, more separated from medicine, delegated to special teachers, doubtless to the advantage and width of scope of these sciences, and to the greater knowledge of them, but I fear there is hereby engendered a tendency to take the student too far afield. . . . It is apt to lead too much to meandering its altitudes, too little to straight going on *terra firma;* too much to pride and obtrusiveness of supposed higher knowledge, too little to reasoning, and too little to power by reasoning upon simple data, and too little to that sort of reasoning which constitutes the basis of common sense. The scientific and the practical, in short, become too much separated. What is needed is a greater regard to that connection between the two which should be maintained through the whole period of study." If these opinions expressed fifteen years ago were correct, they will apply with still greater force to the teaching of today. Let us come to more recent times—especially the last two years.

Let us quote from a physiologist of high repute. Professor Ernest Sterling, of University College, London, during a discussion at the meeting of the British Medical Association at Sheffield in 1908, said: "The tendency for anatomical education to be imparted by professed anatomists has led to increased demands upon the student in the way of accuracy of knowledge. . . . Pharmacology is practically a new science. . . . . The work demanded of a student has practically doubled in amount and is steadily increasing. What is the result? We are trying now to get two pints into a pot that formerly held one. . . . The result is that the student is overburdened from the very beginning of his career. In his first year we try to make him a man of science. To this end we stuff him with facts and absorb the whole of his time in classes, so that he has no leisure for independent thought."

The following extract is taken from a leading editorial in the *British Medical Journal* last April: "Biology as taught by non-medical biologists must go. All the biology a student wants can be given him in his physiological and anatomical courses, and in the study of parasitology and helminthology under the pathologist. Chemistry in the future must be taught by the physiological chemist, and physics by the physiological physicist, by medical men who have gone through the whole training and know the needs and aims of practical medicine. . . . In anatomy great reform is needed, for the size of the present text-books, and the mass of useless detail required, have reached the limit of pedagogic absurdity."

While our college professors are studying methods in medical education, many of our general practitioners are watching the situation with a very deep and intelligent interest. We think the majority of physicians consider it unwise to endeavor to stuff a quart of material into a pint pot. Many of them also believe that our teachers should teach less in order that our learners may learn more. A certain proportion favor Fletcherisation because of their belief that the intellectual pabulum given to our students should be properly digested and thoroughly assimilated.

By a process of evolution the general practitioner frequently develops into a specialist. We have also the ready-made specialist, to whom reference has previously been made. The relationship between the general practitioner and the specialist has been much discussed in the past. Dr. Matthew D. Mann, of Buffalo, read a paper last February on "Dichotomy" or "Dividing Professional Fees." It would appear from what he says that a large proportion of surgeons in the United States are in the habit of giving percentages or commissions to physicians who send them patients, without the knowledge of the latter. I hope it is not necessary to tell members of this Association that such conduct is undignified, unethical and dishonest. It is quite true that the division of fees between the general practitioner and the operating surgeon is frequently or perhaps generally unfair to the former. How can a more fair division be made? We are inclined to think the general practitioners must find that out for themselves. At the present time the relationship between general practitioners and specialists is being considered by a strong committee nominated by the Medical Society of the County of Erie, New York. We shall look forward to their report with much interest.

The general practitioner takes great interest in the work of the specialist. When he goes into a modern hospital theatre while a surgical operation is being performed he beholds something which fills him with wonder and admiration. He asks: "What are these which are arrayed in white robes? and whence came they?" The master of ceremonies answers: "These are they who have discovered something 'more rational' than antiseptic surgery as practised by Lister." The general practitioner does not object to a uniform. The surgeon may wear a nightcap, a mask, a nightgown, mittens and top boots in his well-equipped hospital with all sorts of new apparatus and laboratory appliances if he pleases. There is grave danger, however, that the undue exaltation of modern histrionics may overshadow the real essentials in connection with the prevention of sepsis. We want men of the Lister type to teach our students and practitioners. The wondrous charm of Lister's simplicity in his methods of teaching and operating is one of the most delightful things the world has ever contemplated. Some of our shining lights nowadays, in hospitals and medical societies, appear to aim at giving exhibitions of their skill instead of imparting some practical knowledge to the everyday doctor—knowledge that will help him while working on the side lines or in the backwoods, where theatrical costumes can scarcely come into general use.

When His Majesty our late king came to Canada in 1860 he traveled from the far East as far West as our railway trains could carry him. That far West was Sarnia, in the Province of Ontario. If he had returned twenty-five years later he might have traveled more than two thousand miles further west to a beautiful town called Victoria. There are now in that great western district populous cities and towns in all parts, well-cultivated farms, with an active, intelligent people building up one of the greatest countries in the world.

That great new country has helped this Association very materially during the last twenty years. The crowning result appeared last year when there was held in that modern, beautiful city, Winnipeg, the largest and most successful meeting our Dominion Medical Association has ever known. We slow, sleepy folk of the East respect our brethren of the West because of their ability, we admire them because of their untiring energy, we love them because of their big, warm hearts, we enjoy their generous hospitality beyond expression. We are becoming infected with something akin to their boundless enthusiasm. Especially is this the case in connection with the question of Dominion registration.

The discussion on this subject in Winnipeg was one of the best that have occurred during the last twenty years, and the address delivered by Dr. Thornton, of Deloraine, Manitoba, was one of the best our members have ever heard. He directed our attention to the national side of the question. He told us that "Canada has made great strides toward nationhood in many of the important details of national life, but in the practice of medicine this ideal was no further advanced than in 1866 when Confederation was accomplished. The Provinces were to-day as widely separated as if they flew different flags. There was no such thing as a Canadian physician or a Canadian Medical Association in the broad sense of the terms." We are glad to know that that broad, public-spirited member of our profession, of whom we are so proud, Dr. Thos. G. Roddick, is still taking a very active interest in this question; and we sincerely hope, both for his sake and our own, that his magnificent work will soon meet with the success which it so richly deserves.

This Association is growing not only in numbers, but also in the sphere of its work. We are now considering many matters of vital importance to the people of the whole Dominion, chiefly in the direction of the physician's noblest and most unselfish work—the prevention of disease. We shall have the pleasure this afternoon of learning something respecting the invaluable work accomplished by one of our committees, known as the "Milk Commission," during the past two years, under the able chairmanship of Dr. Chas. J. Hastings.

It would be interesting to give some account of the work done by our Executive Council, the various standing committees, the committee having in charge the establishment of a journal, the local committees, and many individual members in all parts of this big Dominion during the past year. Your President on this occasion, however, cannot find words to describe their work in a fitting manner. Even if he were inclined to undertake such a task the Committee of Arrangements has not given him a sufficient number of hours to accomplish it.

We are all happy now over the present condition of our Association. We are filled with hope for the future. We are becoming national in the true sense of the term. May I add—we are growing more imperialistic. We really want not only Dominion registration, but also reciprocity with the profession of our dear mother country. Although we are plunged in grief over the appalling calamity that has befallen our great Empire, our wish, our song, our hymn, our prayer is still—God save the King.

